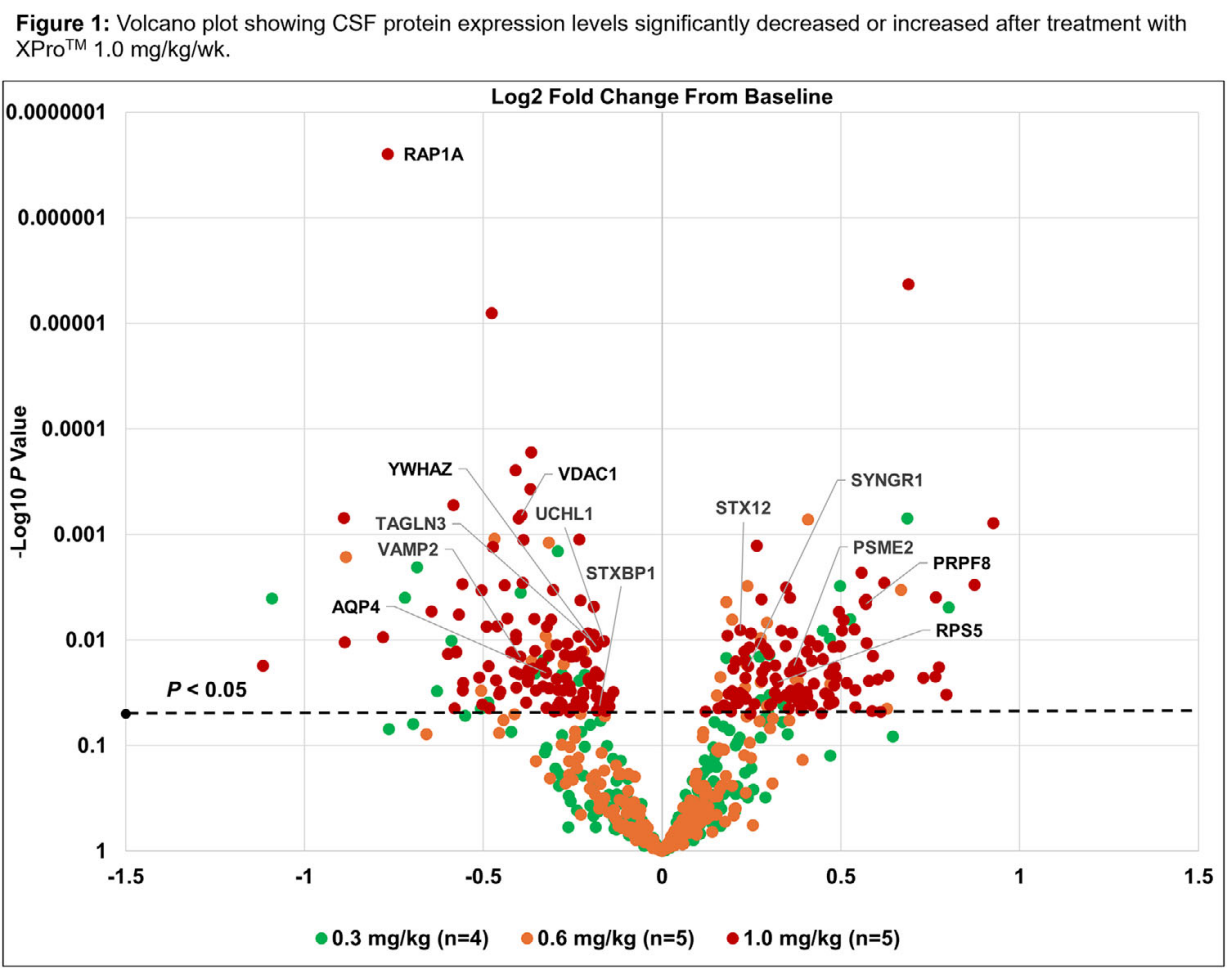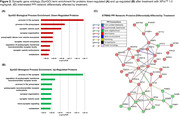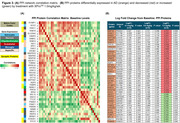# Dose‐related modulation of the synaptic proteome after short‐term treatment with XPro1595 for Alzheimer’s disease

**DOI:** 10.1002/alz.095343

**Published:** 2025-01-09

**Authors:** Parris Pope, Ian Pike, CJ Barnum, RJ Tesi

**Affiliations:** ^1^ INmune Bio, Boca Raton, FL USA; ^2^ Proteome Sciences plc, London United Kingdom

## Abstract

**Background:**

XPro1595 (XPro^TM^) is a brain‐penetrant, recombinant protein variant of human tumor necrosis factor (TNF) rationally designed to selectively neutralize only the soluble, pro‐inflammatory form of the cytokine (solTNF). An unbiased proteomic analysis of CSF samples from an open‐label, phase‐1b study (NCT03943264) in patients with Alzheimer’s disease (AD) was conducted to assess for pharmacodynamic activity and disease‐specific target engagement.

**Method:**

Patients with AD (n = 20) were treated for 12‐weeks with one of three doses of XPro^TM^: 0.3 (n = 5), 0.6 (n = 6) or 1.0 mg/kg/wk (n = 9). Tandem‐mass tag mass spectrometry (TMT‐MS) proteomics was performed on CSF, with lysates from three postmortem AD brains (Braak Stage IV‐VI) as control samples. Protein levels were recorded as Log2 ratios (sample/reference), with two‐sided t‐tests for determination of significant change from baseline. Differential abundance (Metascape and SynGO) and proteomic pathway (STRING) analyses were used to identify gene ontology (GO) biological processes (GOBPs) and interrelated clusters dose‐dependently affected by treatment. Due to the small sample size and exploratory nature of the study, the threshold for determination of informative values was set at *P*<0.05 (nominal), with no correction for multiple comparisons.

**Result:**

CSF samples from fourteen patients were available for analysis: 0.3 (n = 4), 0.6 (n = 5), and 1.0 mg/kg/wk (n = 5). In total, 29,607 peptides associated with 3,632 distinct protein groups were quantified in all samples. CSF levels of 221 proteins significantly increased (n = 111) or decreased (n = 116) after treatment with the 1.0 mg/kg/wk dose of XPro^TM^ (Figure 1). GOBP and STRING pathway analyses showed high enrichment for synaptic proteins (24%) and identified a protein‐protein interaction (PPI) network of 41 proteins affected by treatment in a dose‐related manner (Figure 2). Analysis of baseline levels by PPI group (decreased or increased) showed positive within‐group intercorrelations (*r* > 0.535, *P*<0.05), with similarly negative correlations across the two groups, thus confirming interrelatedness in this sample (Figure 3A). Thirty‐two (78%) of the PPI proteins were also found to be differentially expressed in AD (Figure 3B).

**Conclusion:**

Synaptic dysfunction is associated with both the clinical symptoms and core pathologies of AD. These findings provide further evidence of disease‐specific and dose‐related target engagement for XPro^TM^ in AD.